# Whole-genome sequence analysis of SFTS bunyavirus in Huzhou, China

**DOI:** 10.1371/journal.pone.0318742

**Published:** 2025-02-11

**Authors:** Deshun Xu, Lei Ji, Xiaofang Wu, Liping Chen

**Affiliations:** Huzhou Center for Disease Control and Prevention, Huzhou, China; Instituto Butantan, BRAZIL

## Abstract

Severe fever with thrombocytopenia syndrome (SFTS), a tick-borne emerging infectious disease caused by SFTS virus (SFTSV), is a growing public health threat due to its high mortality rate. To understand the genomic characteristics of SFTSV samples isolated in Huzhou, China, the full-length genomes of Huzhou SFTSV isolates obtained between February 1, 2019 and December 30, 2023 were sequenced, and the gene loci, evolution, and sequence identity of the genome sequences were analyzed using MEGA. The complete genome sequences of seven SFTSV samples were obtained successfully. The full-length genome of each isolate was 11 490 bp in length, composed of a large (L) segment of 6368 bp, medium (M) segment of 3378 bp, and small (S) segment of 1744 bp. The SFTSV samples isolated in Huzhou belonged to multiple genotypes, but were mainly of type D. Each subtype showed nucleotide sequence and amino acid sequence identities of more than 93.67% and 97.18%, respectively, with the syngeneic human host reference strain and more than 93.67% and 97.76%, respectively, with the syngeneic tick-derived host reference strain. Nucleotide sequence analysis of SFTSV isolates from Huzhou showed mutations in genes on all three segments, with those on the M segment showing the highest mutation rate. The nucleotide variations were mainly base transversions. Further studies of the distribution of SFTSV genotypes, sites of nucleotide mutations, and amino acid variations are required.

## Introduction

Fever with thrombocytopenia syndrome (SFTS) is a newly emerging insect-borne zoonotic disease, which was first discovered by the Chinese Center for Disease Control and Prevention in 2010 [1,2]. The disease is caused by a novel RNA virus known as Bandavirus dabieense, which belongs to the genus *Phlebovirus* within the family Bunyaviridae [3,4]. SFTSV infection occurs mostly in forests, grasslands rich in vegetation, and densely vegetated mountainous and hilly areas [5]. Most common in spring and summer, the disease affects predominantly middle-aged and older adults tending farms in hilly areas. The major clinical symptoms and laboratory findings of SFTS include fever, thrombocytopenia, gastrointestinal symptoms, multifunctional organ damage, leukopenia, and elevated levels of serum hepatic enzymes. The lack of effective vaccines and treatment options contributes to the relatively high mortality rate of SFTS. The mortality rate of SFTS currently ranges from 5%–30% in East Asia, with patients usually dying of multiple organ failure [6,7]. SFTSV infection was first reported in China, with additional cases subsequently confirmed in Japan and Korea [8]. Recently, molecular and serological detection of SFTSV in both humans and animals has been reported in Vietnam, Taiwan, Thailand, Pakistan, and Myanmar [9–13]. These reports indicate the circulation of SFTSV in other South Asian countries as well as the endemic countries, such as China, Japan, and Korea. The World Health Organization (WHO) ranked SFTS as the disease with the highest research priority in 2017 [14].

Due to the life cycle of SFTSV in nature, the genotype and evolution of SFTSV are closely correlated with the geographical region [15]. In evolutionary trees, SFTSV strains are categorized into two clades, the Chinese lineage (consisting of genotypes A-F, except B), which includes most strains from China and a few from South Korea, and the Japanese lineage (consisting of genotype B), which includes strains from South Korea and Japan and a few from China [16,17]. The most prevalent genotype in Japan and South Korea is subgenotype B-2, followed by B-3 and B-1 [18–20], whereas genotypes F, A, and D are the most common in China [21–23]. Vietnam has identified S segments of genotype B-3 [24].

SFTSV is spherical in shape with a diameter of 80–120 nm and has an RNA genome composed of three independent gene segments designated as large (L), medium (M), and small (S). The L segment encodes the RNA-directed RNA polymerase (RdRp), the M segment encodes the envelope glycoproteins (GPs) Gn and Gc that play important roles in receptor binding and entrance into cells, and the S segment is an ambisense RNA encoding a nucleocapsid protein (NP) and a nonstructural protein (NS) that interacts with the interferon signaling pathway and plays an important role in evading innate immunity [25,26]. Similar to other trisegmented RNA viruses, SFTSV has high substitution rates and recombinant and reassorted strains, leading to widespread geographical co-circulation of viral strains [27,28].

Ticks are among the primary host vectors for SFTSV [29], and human-to-human transmission has been reported in some clusters and patients [30,31]. SFTSV has been reported to be transmitted through blood contact, droplet contact [32], and aerosolized droplets [33]. Genotyping is a crucial method for virus classification and research. Through genotyping, researchers can ascertain the epidemiological characteristics of the virus, including geographical distribution, infected populations, and epidemic trends. It also enables the determination of genetic relationships between different strains, which is essential for vaccine development, epidemiological investigations, and studies on virus evolution. Furthermore, genotyping can evaluate the pathogenicity and virulence of viruses, offering valuable insights for disease prevention and treatment. Additionally, it provides a foundation for virus monitoring and control, including the development of vaccine strategies and the implementation of prevention and control measures. In this study, whole-genome sequencing analysis of SFTSV isolates obtained in Huzhou, China, between February 1, 2019 and December 30, 2023 was performed to understand the genetic characteristics and molecular epidemic trends of SFTSV in this region, and to provide a scientific basis for SFTSV epidemic surveillance and infectious disease prevention as well as a theoretical basis for vaccine and drug research and development.

## Materials and methods

### Ethics statement

This study was approved by the Human Research Ethics Committee of Huzhou Center for Disease Control and Prevention (approval number: HZ2019006). This study was performed as part of a routine laboratory-based investigation and included no human experimentation. The only human materials used were blood specimens that had been sent to our laboratory for routine virological diagnosis. All patients or their guardians provided written informed consent for research use of blood specimens.

### Clinical samples and nucleic acid extraction

A total of 101 serum samples were collected from patients suspected of having SFTS who exhibited symptoms such as high fever ( ≥38°C), vomiting, diarrhea, thrombocytopenia, and leukocytopenia. And sent to Huzhou Center for Disease Control and Prevention for SFTS nucleic acid detection during the period between February 1, 2019 and December 30, 2023. Additionally, 200 mL samples were absorbed, and viral RNA was extracted using the QIAamp Virus RNA Minikit (Qiagen, Hilden, Germany). The RNA was ultimately eluted in 50 µ L of eluent..

### Whole-genome sequencing

The presence of SFTSV genome was detected by Real-time quantitative reverse transcription-polymerase chain reaction (RT-PCR). An RNA whole-genome sequencing library was constructed with an NGSmaster Pathogen Metagenomic One-Stop Library-Building Kit (Jieyi Biotechnology, Hangzhou, China) and DNA/RNA extraction and library building kit (Jieyi Biotechnology) for detection of SFTSV virus nucleic acid. The genome libraries were sequenced using a NextSeq 550 sequencer (Illumina, San Diego, CA, USA). All operations were carried out in accordance with the respective manufacturer’s instructions.

### Sequence analysis

Quality control analysis of the sequence data was performed using FastQC (v0.11.9), fastp (v0.23.2) was used to remove low-quality data, and the new bunyavirus strains Zhao (KF374682–KF374684) were used as the reference genomes for comparisons using bwa (v0.7.17), achieving an alignment rate of 98.16%–99.24%. Consensus sequences were produced using ivar, with the whole genome length being 11,490 and a sequencing depth exceeding 8000x. The sequences of the new bunyavirus reference strains of each genotype were obtained from the National Center for Biological Information (NCBI) GenBank database. The sequences were edited and multiple alignment was performed using ClustalW in BioEdit (v7.0.9.0). BioAider (v1.527) was used for analysis of nucleotide and amino acid sequence identity. The intra- and interspecies distances among the new bunyavirus isolates, human-derived reference strains, and tick reference strains were calculated using the Kimura 2 parameter (K2P) two-parameter model in MEGA (v7.0) for genetic distance analysis and phylogenetic analysis. Following the comparison of the translated protein sequence of the new bunyavirus isolate with reference strains derived from humans and ticks, the mutation sites were examined. The Phylogeny function utilized a Kimura 2-parameter model in conjunction with the Neighbour-Joining method and a Bootstrap method with 1000 replications. The three gene sequences of the new bunyavirus isolate, the homologous human-derived reference strain, and the homologous tick reference strain were aligned with new bunyavirus isolates from other regions in GenBank to construct a phylogenetic tree.

### Nucleotide sequence accession numbers

The sequences obtained in this study have been deposited in GenBank with accession numbers PP488475–PP488495.

## Results

### SFTSV samples sequence information

A total of 10 SFTSV virus nucleic acid-positive samples were detected from among 101 serum specimens from patients with suspected SFTSV infection. The whole genomes of seven SFTSV virus samples were obtained by second-generation sequencing. These whole-genome sequences were divided into three segments designated as the L segment, M segment, and S segment. The results were compared with the reference genome (KF374682–KF374684). The L segment had a total length of 6368 nucleotides and contained an open reading frame (ORF) consisting of 17–6271 nucleotides at the 5′ end of the coding RNA (cRNA) encoding a predicted product of 2084 amino acids. The M fragment had a total length of 3378 nucleotides and contained an ORF of 19–3240 nucleotides at the 5′ end of the cRNA encoding a predicted product of 1073 amino acids. The S fragment was an ambisense RNA 1744 nucleotides in length with two ORFs in opposite orientations separated by an intergenic region of 54 nucleotides: one ORF of 29–910 nucleotides at the 5′ end of the cRNA encoding a predicted product of 293 amino acids (NS) and another ORF of 1702–965 nucleotides in the opposite orientation at the 3′ end encoding a product of 245 amino acids (NP).

### Phylogenetic analysis

**Fig 1:**
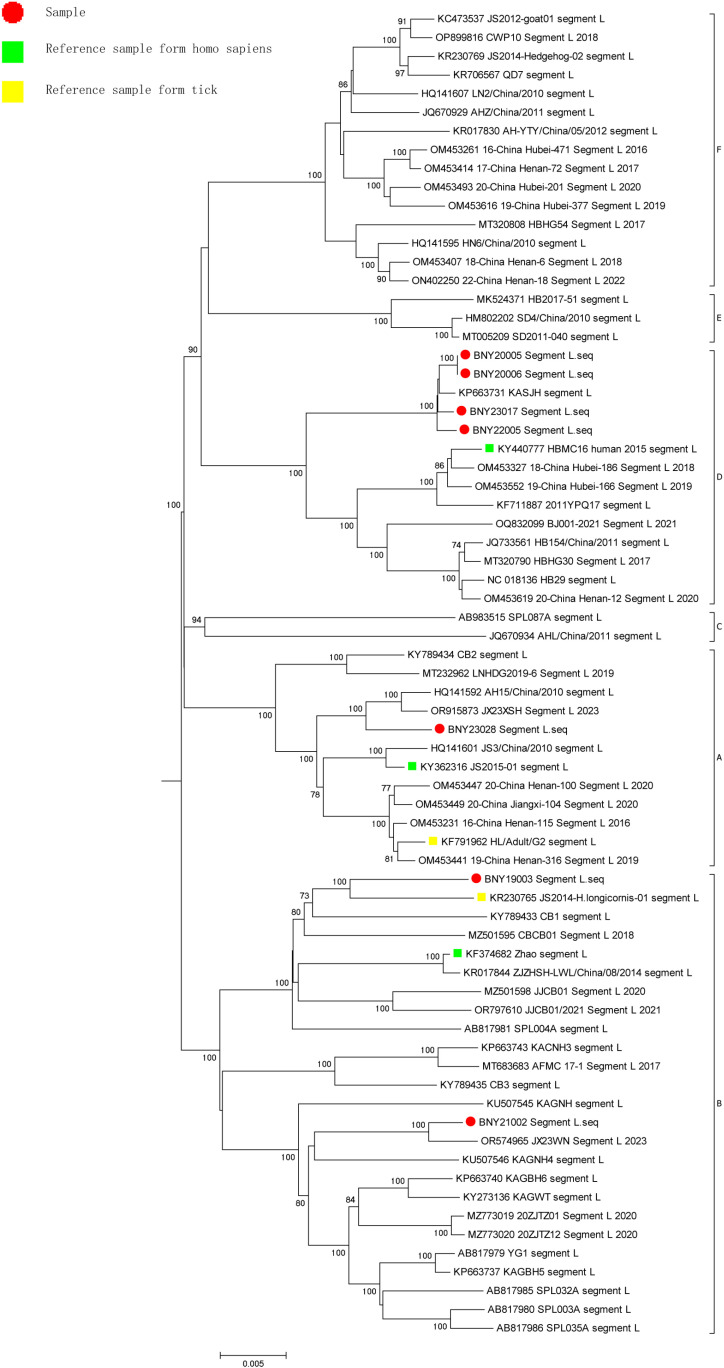
Neighbor-joining tree of the L segment.

**Fig 2:**
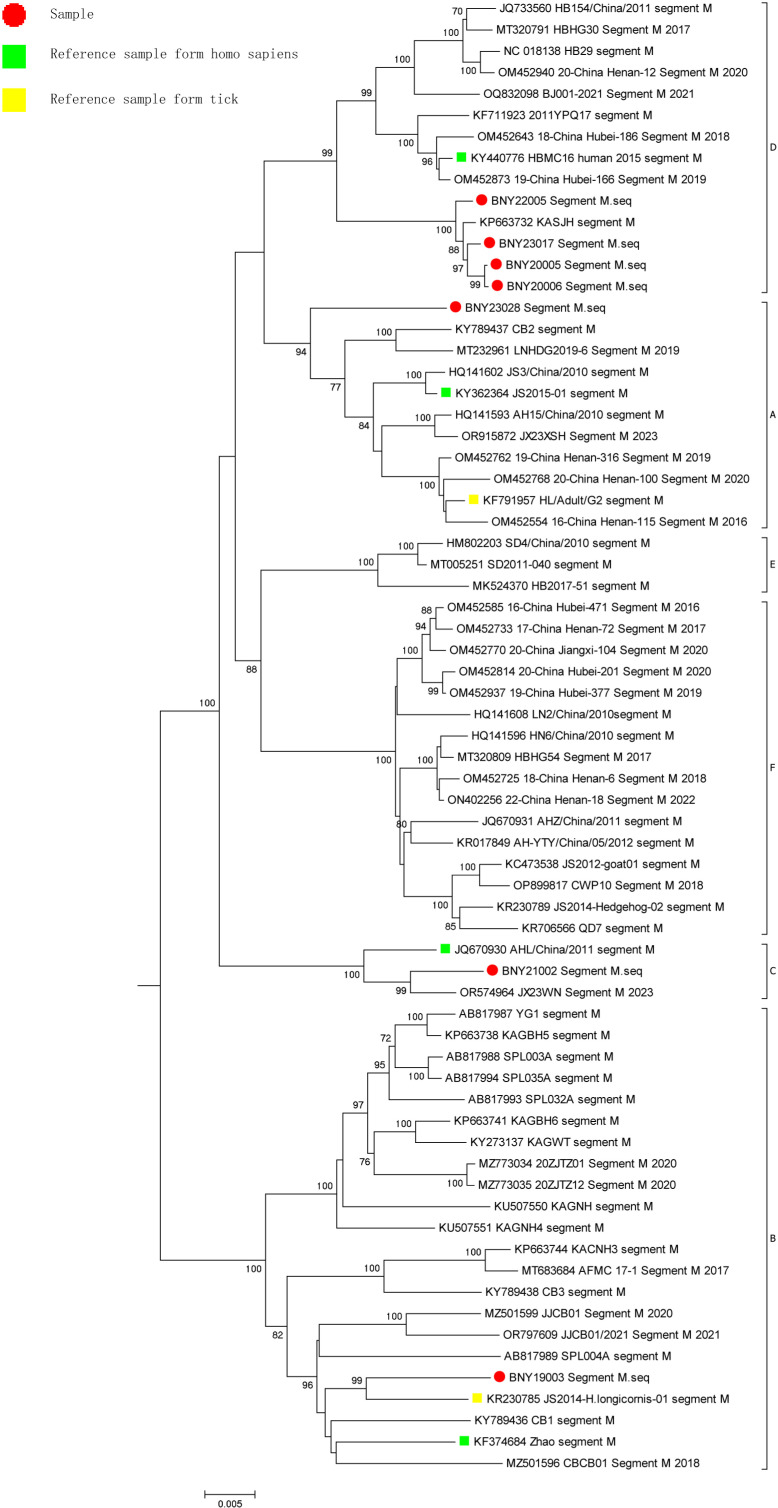
Neighbor-joining tree of the M segment.

**Fig 3:**
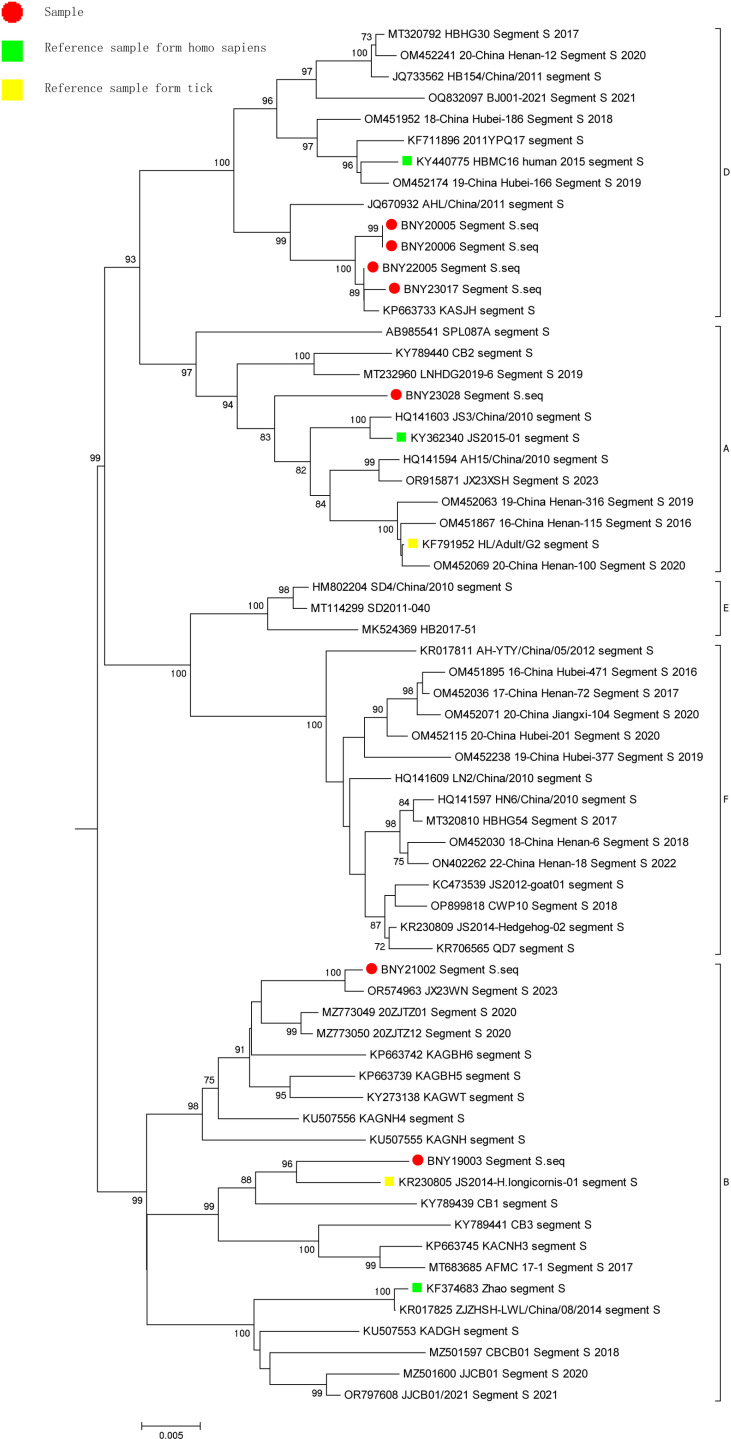
Neighbor-joining tree of the S segment.

A phylogenetic tree was constructed between the SFTSV isolates and the gene sequences of representative strains of SFTSV from other regions of China or internationally in NCBI using MEGA (v7.0.14). Analysis of L segment gene sequence identity showed that one of the seven SFTSV samples belonged to the genotype A branch with a self-spread value of 100. Two SFTSV samples belonged to the genotype B clade with a self-spread value of 100, and the two samples were divided into two clades. Four SFTSV samples belonged to the genotype D branch with a self-spread value of 100 and formed a cluster with a self-spread value of 100 (Fig 1). Analysis of M segment gene sequence identity showed that one of the seven SFTSV samples belonged to the genotype A branch with a self-spread value of 94. One SFTSV sample belonged to the genotype B branch with a self-spread value of 100. One SFTSV sample belonged to the genotype C branch with a self-spread value of 100. Four SFTSV sample belonged to the genotype D branch with a self-spread value of 99 and formed a cluster with a self-spread value of 100 (Fig 2). Analysis of S segment gene sequence identity showed that one of the seven SFTSV samples belonged to the genotype A branch with a self-spread value of 97. Two SFTSV samples belonged to the genotype B branch with a self-spread value of 99 and were divided into two clades. Four SFTSV samples belonged to the genotype D branch with a self-spread value of 100 and formed a cluster with a self-spread value of 99 (Fig 3).

### Homology analysis

Analysis of genes on the L and S fragments showed that the seven SFTSV isolates from Huzhou belonged to genotypes A, B, and D. Analysis of the M segment genes Gn and Gc divided the isolates into genotypes A, B, D, and C. The seven Huzhou SFTSV isolates showed 95.91%–100% and 99.47%–100% nucleotide and amino acid identity in the L segment gene RdRp, respectively. The M segment genes showed 93.73%–99.97% and 98.04%–99.91% nucleotide and amino acid identity, respectively. On the S segment, the levels of nucleotide and amino acid identity were 94.67%–100% and 97.96%–100%, respectively, for the NS gene and 94.99%–100% and 99.59%–100%, respectively, for the NP gene (S1 Table).

The L segment gene RdRp showed 96.23%–98.31% and 99.52%–99.81% nucleotide and amino acid identity with the genotype A human host reference strain (KY362316.1), respectively. The M segment genes showed 94.01%–97.45% and 98.51%–98.7% nucleotide and amino acid identity with the genotype A human host reference strain (KY362364.1), respectively. The levels of nucleotide and amino acid identity with the genotype A human host reference strain (KY362340.1) were 94.44%–97.28% and 98.3%–99.66%, respectively, for the NS gene and 95.39%–98.51%, and 99.59%–100%, respectively, for the NP gene on the S segment. The L segment gene RdRp showed 95.96%–97.51% nucleotide identity and 99.57%–99.86% amino acid identity with the genotype B human host reference strain (KF374682.1). The M segment genes showed 93.67%–97.3% nucleotide identity and 98.04%–99.26% amino acid identity with the genotype B human host reference strain (KF374684.1). On the S segment, the levels of nucleotide and amino acid identity with the genotype B human host reference strain (KF374683.1) were 94.44%–97.05% and 98.64%–100% for the NS gene and 94.99%–95.8% and 99.19%–99.59% for the NP gene, respectively. The M segment genes showed nucleotide and amino acid sequence identity of 94.57%–98.04% and 98.32%–99.72% with the human host reference strain JQ670930.1 and 94.01%–97.39% and 98.14%–98.98% with the genotype D human host reference strain (KY440776.1), respectively. The levels of nucleotide and amino acid identity with the genotype D human host reference strain (KY440775.1) were 94.67%–96.6% and 97.28%–97.96% for the NS gene and 95.12%–98.64% and 99.59%–100% for the NP gene on the S segment, respectively (S1 Table).

The L segment gene RdRp showed nucleotide identity of 96.08%–98.19% and amino acid identity of 99.52%–99.81% with the genotype A tick-derived host reference strain (KF791962.1). The M segment genes showed nucleotide and amino acid identity of 93.76%–97.3% and 98.32%–98.51%, respectively, with the genotype A tick-derived host reference strain (KF791957.1). On the S segment, the levels of nucleotide and amino acid identity with the genotype A tick-derived host reference strain (KF791952.1) were 94.56%–97.39% and 97.96%–99.32% for the NS gene and 94.85%–98.92% and 99.59%–100% for the NP gene, respectively. The L segment gene RdRp showed nucleotide identity of 95.81%–98.19% and amino acid identity of 99.42%–99.76% with the genotype B tick-derived host reference strain (KR230765.1). The M segment genes showed nucleotide identity of 93.67%–97.83% and amino acid identity of 97.95%–99.35% with the genotype B tick-derived host reference strain (KR230785.1). The levels of nucleotide and amino acid identity with the genotype B tick-derived host reference strain (KR230805.1) were 94.67%–98.07% and 98.98%–99.66% for the NS gene and 95.66%–98.92% and 99.59%–100% for the NP gene on the S segment, respectively (S1 Table).

### Amino acid sequence analysis

Huzhou isolate BNY23028 had variations in seven amino acid sites in Lsegment compared with

both the genotype A human reference strain (KY362316.1) and with the genotype A tick-derived host reference strain (KF791962.1). BNY19003 and BNY21002 had six and eight amino acid variations in Lsegment compared with the genotype B human reference strain (KF374682.1) and with the genotype B tick-derived host reference strain (KR230765.1). BNY20005, BNY20006, BNY22005, and BNY23017 had 15 amino acid variations in Lsegment compared with the genotype D human reference strain (KY440777.1) (Table 1) (S2 Table).

**Table 1:** Changes in L segment Antigenic Sites.

L - amino acid site	2	109	128	161	167	243	307	314	381	397	418	448	449	479	566	586	703	710	818	1199	1241	1353	1367	1433	1447	1500	1651	1678	1711	1717	1744	1808	1825	1837	1894	2070
BNY19003	N	L	S	E	F	G	K	S	V	E	S	E	T	I	Y	D	L	M	A	I	A	N	E	A	I	I		S	I	I	S	I	K	V	M	C
BNY20005	N	M	S	E	F	G	K	G	V	E	S	E	T	V	Y	D	L	M	A	I	A	N	E	T	I	I	I	S	I	V	S	I	K	V	M	C
BNY20006	N	M	S	E	F	G	K	G	V	E	S	E	T	V	Y	D	L	M	A	I	A	N	E	T	I	I	I	S	I	V	S	I	K	V	M	C
BNY21002	N	M	S	E	F	G	R	S	I	E	S	E	T	V	Y	D	L	M	A	I	A	N	E	A	I	I	I	S	I	I	C	I	K	V	M	C
BNY22005	N	M	S	E	F	G	K	G	V	E	S	E	T	V	Y	D	L	M	A	I	A	N	E	T	I	I	I	S	I	V	S	I	K	V	M	F
BNY23017	N	M	S	E	F	G	K	G	V	E	N	E	T	V	Y	D	L	M	V	I	A	N	E	T	I	I	I	S	I	V	S	I	K	V	M	C
BNY23028	N	M	S	E	F	G	K	G	V	E	S	E	A	V	Y	D	L	M	A	I	T	S	G	T	I	I	I	S	S	V	S	I	K	V	M	C
KY362316.1-human-A	D	M	S	G	F	G	K	G	V	E	S	E	A	V	Y	D	L	M	A	I	A	N	E	T	I	M	I	S	I	V	S	I	K	V	M	C
KF374682.1-human-B	N	M	S	E	F	G	K	S	V	E	S	E	T	V	Y	D	L	M	A	I	A	N	E	A	I	I	M	S	I	I	S	I	K	V	M	C
KY40777.1-human-D	N	M	S	E	L	G	K	G	V	D	S	E	T	V	F	D	F	I	A	V	A	N	E	T	V	I	I	S	I	V	S	I	R	I	I	C
KF791962.1_haemaphysalis longicornis_A	N	M	T	E	F	R	K	G	V	E	S	D	A	V	Y	E	L	M	A	I	A	N	E	T	I	I	I	S	I	V	S	I	K	V	M	C
KR230765.1_haemaphysalis longicornis_B	D	M	S	E	F	G	K	S	I	E	S	E	T	V	Y	D	L	M	A	I	A	N	E	A	I	I	I	A	I	I	S	V	K	V	M	C

Huzhou isolate BNY23028 had 15 and 17 amino acid variations in M segment compared with the genotype A human reference strain (KY362364.1) and the genotype A tick-derived host reference strain (KF791957.1), respectively. Isolate BNY19003 had eight amino acid variations in M segment compared with the genotype B human reference strain (KF374684.1). Compared with the genotype B tick-derived host reference strain (KR230785.1), there were seven amino acid variants in M segment in BNY19003. The Huzhou isolates BNY20005, BNY20006, BNY22005, and BNY23017 had 12, 13, 12 and 13 amino acid variations in M segment compared with the genotype D human reference strain (KY440776.1), respectively. BNY21002 had three amino acid variations in M segment compared with the genotype C human reference strain of (JQ670930.1). (S5 XLSX and S3 Table).

BNY23028 had three amino acid variations (A15V, R130K, and V229D) in NS protein and none in NP protein compared with the genotype A human reference strain (KY362340.1). BNY23028 had four amino acid substitutions (A15V, R130K, S143G, and D276N) in NS protein and none in NP protein compared with the genotype tick-derived host reference strain (KF791952.1). Isolates BNY19003 and BNY21002 had two amino acid substitutions (G242E and T272A) in NS protein and one amino acid substitution (R95K) in NP protein compared with genotype B human reference strain (KF374683.1). BNY19003 had seven amino acid substitutions in M segment compared with the genotype B tick-derived host reference strain (KR230785.1). BNY19003 had three amino acid substitutions (G242E, A243V, and T272A) and BNY21002 had one substitution (A243V) in NS protein and no substitutions in NP protein compared with the genotype B tick-derived host reference strain (KR230805.1). BNY20005, BNY20006, BNY22005, and BNY23017 had six amino acid substitutions (L16M, F30Y, R130K, T172I, V243A, and P245Q) in NS protein and none in NP protein compared with the genotype D human reference strain (KY440775.1) (S4 Table).

### Recombination analysis

**Fig 4.**
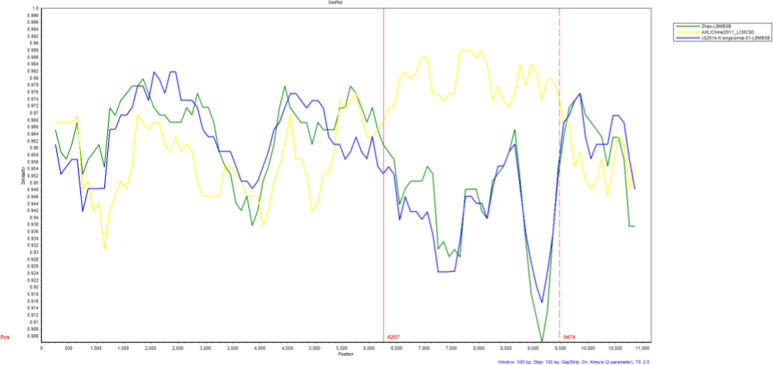
Recombination analysis of BNY21002.

The ORFs from segments L, M, and S of seven samples of SFTSV were combined into a single linear fragment and analyzed for recombination events with each reference strain using T-RECS software. Consistent with the phylogenetic analysis outlined above, the results showed that isolate BNY21002 had the greatest similarity with genotype B variants in the L and S segments and the highest similarity with genotype C variants in the M segment. No recombination was found in the remaining six samples (Fig 4).

## Discussion

Huzhou is located in the north subtropical monsoon climate zone of China, with both rain and heat in the same season, abundant precipitation, and a mild and humid climate. The terrain fluctuates markedly with large differences in altitude from mountainous in the west to alternating low hills and plains in the northwest. These conditions are suitable for ticks and other viral vectors. There have been a number of sporadic cases of SFTSV infection in Huzhou in recent years, which was also confirmed by examination of serum samples from patients with suspected SFTSV infection in 2019–2023.

Previous studies have shown clear regional differences in the distribution of SFTSV genotypes. The nomenclature for SFTSV genotypes is based on the country of origin, e.g., C1–C5 for Chinese lineages and J1–J3 for Japanese lineages. Fu *et al.* [34] proposed a new nomenclature for SFTSV strains, which divides the viruses into six clades referred to as genotypes A to F. SFTSV genotypes A, D, and F are the most commonly detected genotypes in mainland China, while genotype B is most commonly found circulating in South Korea and Japan [35]. In China, the number of patients with SFTSV infection is increasing, and various genotypes have been detected since 2010. New SFTSV genotypes, reassortments, and recombinant isolates have been identified by domestic researchers’ analysis of SFTSV molecular epidemiological. In this study, the L, M, and S segments of seven SFTSV samples isolated in Huzhou were used to construct molecular phylogenetic trees. The analysis showed that the SFTSV types present in the region are diverse, including genotypes A, B, and D, among which D was the main type (four samples). This was consistent with the fact that D is one of the main epidemic strains in mainland China [35], but inconsistent with the results of Hangzhou and Zhoushan studies showing regional differences in the prevalence of SFTSV [36,37]. It is worth noting that the isolate BNY21002 had the highest degree of similarity with genotype B on the L and the S segments, but the highest degree of similarity with genotype C in the M segments. Recombination between this isolate and the reference strains of each genotype was analyzed using T-RECS, and the results showed that BNY21002 was a recombinant strain. In this study, seven SFTSV samples from Huzhou showed high degrees of identity across each gene type (A, B and D type), with nucleotide and amino acid sequence identity exceeding 90%, respectively, consistent with previous reports [38,39]. The levels of nucleotide and amino acid sequence identity of each type reached 93.67% and 97.18% in comparison with the human host reference strain of the corresponding genotype, and 93.67% and 97.76% in comparison with tick-derived host reference strain of the corresponding genotype, respectively, similar to the results reported previously [39]. Isolates from patients’ blood samples and ticks captured from the areas where these patients lived were reported to show nucleotide and amino acid sequence identity as high as 99% [40]. The above reports suggest that ticks, as intermediate hosts, play an important role in the transmission of SFTSV.

Determination of the nucleotide sequence of the virus genome, analysis of amino acid variations, and determination of the phylogenetic relationships between the sequences allows monitoring of changes in antigenic sites, receptor binding sites, and amino acid sites of viral functional proteins, provides insights into its evolution and trends of variation, and forms a basis for the development of prevention and control measures [41,42]. In this study, we found that the nucleotide sequences of seven SFTSV isolates from Huzhou had variations in proteins encoded on the L, M, and S segments, with the highest mutation rate in the M segment. Compared with the reference strain of the corresponding genotype, BNY23028 had the highest mutation rate (15 amino acid substitutions in M segment) and 17 amino acid variations in M segment compared with the tick-derived host reference strain of the corresponding genotype. This high mutation rate in the M segment may have been because it encodes the envelope precursor protein of the virus, which serves as a significant immunogen. During the binding process between the virus and the host, the selection pressure is heightened, potentially leading to a faster evolutionary rate compared to the L and S segments. Further analysis showed that the nucleotide sequences of SFTSV isolates tended to involve transitions between bases, suggesting that there may be a single base bias in the nucleotide variation of SFTSV strains that is common in the process of virus mutation.

RNA viruses are characterized by a high mutation rate and significant recombination potential, resulting in considerable genomic heterogeneity [43]. Reassortment events are prevalent among viruses with segmented genomes, including bunyaviruses [44]. Natural reassortment of SFTSV has been documented from various sources, such as human patients, ticks, and animals [45,46]. Additionally, recombination represents another potential mechanism driving SFTSV evolution [36]. In this paper, we further demonstrate that the isolate BNY21002 from 2021 originated from human patients. Notably, the recombination events of SFTSV in Huzhou predominantly occurred in the M segment, with no recombination observed in the L and S segments. This observation suggests that this viral lineage may be experiencing active evolution and adaptation, potentially generating adaptive mutations while eliminating deleterious ones through exchanges with other genomic sites. Such changes may influence the virus’s antigenicity, tropism, and pathogenicity. However, the implications of these recombination events for epidemic potential and their association with disease severity warrant further investigation

## Conclusion

Although the present study had limitations in terms of the number of samples analyzed, the findings provided insight into the genetic characteristics and genotype distribution of SFTSV samples in Huzhou, China, and provide a scientific basis for SFTS epidemic surveillance and preventive measures. In the future, we will actively conduct SFTS surveillance and enhance collaboration between hospitals and the Centers for Disease Control and Prevention to better understand the pathogenicity of the virus and the effects of amino acid mutations on the function of SFTSV.

## Supporting information

S1 Table Nucleotide homology and amino acid homology analysis of L, M and S segment. (DOCX)

S2 Table Analysis of amino acid variation in L segment of the genotype A, B and D. (DOCX)

S3 Table Analysis of amino acid variation in M segment of the genotype A, C and D. (DOCX)

S4 Table Analysis of amino acid variation in S segment of the genotype A, B and D. (DOCX)

S5 Table Changes in M segment Antigenic Sites. (XLSX)
