# Supplementary material for: Whole-genome sequence analysis of SFTS bunyavirus in Huzhou, China
Source: PLoS One. 2025 Feb 11;20(2):e0318742. doi: 10.1371/journal.pone.0318742 (PMC11813122; doi:10.1371/journal.pone.0318742)
Supplement: S1 Table — (DOCX) [file pone.0318742.s001.docx]

**Nucleotide homology and amino acid homology analysis of L segment**

AA

**Nucleotide homology and amino acid homology analysis of M segment**

AA

**Nucleotide homology and amino acid homology analysis of S(NS) segment**

AA

**Nucleotide homology and amino acid homology analysis of S(NP) segment**

AA
