# Supplementary material for: Whole-genome sequence analysis of SFTS bunyavirus in Huzhou, China
Source: PLoS One. 2025 Feb 11;20(2):e0318742. doi: 10.1371/journal.pone.0318742 (PMC11813122; doi:10.1371/journal.pone.0318742)
Supplement: S2 Table — (DOCX) [file pone.0318742.s002.docx]

**Analysis of amino acid variation in L segment of the genotype A**

**Analysis of amino acid variation in L segment of the genotype B**

**Analysis of amino acid variation in L segment of the genotype D**
